# MoS_2_ and Fe_2_O_3_ co-modify g-C_3_N_4_ to improve the performance of photocatalytic hydrogen production

**DOI:** 10.1038/s41598-022-07126-2

**Published:** 2022-02-28

**Authors:** Yan Zhang, Junfen Wan, Chunjuan Zhang, Xuejun Cao

**Affiliations:** State Key Laboratory of Bioreactor Engineering, East China University of Science and Technology, 130 Meilong Rd., Shanghai, 200237 China

**Keywords:** Catalysis, Energy, Photochemistry

## Abstract

Photocatalytic hydrogen production as a technology to solve energy and environmental problems exhibits great prospect and the exploration of new photocatalytic materials is crucial. In this research, the ternary composite catalyst of MoS_2_/Fe_2_O_3_/g-C_3_N_4_ was successfully prepared by a hydrothermal method, and then a series of characterizations were conducted. The characterization results demonstrated that the composite catalyst had better photocatalytic performance and experiment results had confirmed that the MoS_2_/Fe_2_O_3_/g-C_3_N_4_ composite catalyst had a higher hydrogen production rate than the single-component catalyst g-C_3_N_4_, which was 7.82 mmol g^−1^ h^−1^, about 5 times higher than the catalyst g-C_3_N_4_ (1.56 mmol g^−1^ h^−1^). The improvement of its photocatalytic activity can be mainly attributed to its enhanced absorption of visible light and the increase of the specific surface area, which provided more reactive sites for the composite catalyst. The successful preparation of composite catalyst provided more channels for carrier migration and reduced the recombination of photogenerated electrons and holes. Meanwhile, the composite catalyst also showed higher stability and repeatability.

## Introduction

With the rapid development of the social economy, human demand for energy is increasing. Traditional fossil energy is non-renewable, and the extensive use of fossil resources brings a serious crisis to the environment^1–3^. Therefore, it is urgent to find a kind of alternative energy to replace fossil energy. Hydrogen as a clean and renewable energy has received widespread attention. Photocatalytic hydrogen production is a technology that uses solar energy to produce hydrogen, exhibits broad prospect in solving resource and environmental pollution problems^4^. However, in the current photocatalysis research, the semiconductor catalysts show a low rate of hydrogen generation due to their various defects and cannot be widely used. Therefore, the development of efficient and stable photocatalysts has become a hot spot in current research.

In the past few decades, scholars have devoted themselves to finding semiconductor catalytic materials with high catalytic performance, semiconductor materials have been extensively explored, such as TiO_2_^5–7^, CdS^8–10^, ZnO^11–13^ and g-C_3_N_4_^14–16^. Among the various semiconductor catalysts that have been currently developed and researched, g-C_3_N_4_, as a non-metal semiconductor material, has attracted wide attention because of its suitable band gap, good stability, and visible light response. However, its small specific surface area and strong photo-generated electron–hole recombination ability hinder its hydrogen production performance^17–19^. Many methods have been adopted in the current research to improve the performance of the semiconductor catalyst g-C_3_N_4_ and increase its hydrogen production rate. For example, using doped metal and non-metal elements to accelerate the separation of charges^20–23^, changing the structure of g-C_3_N_4_ to increase its specific surface area^24^, forming a heterojunction with other materials^25–27^, or supporting cocatalysts to accelerate electron transfer ^28^.

Hematite (α-Fe_2_O_3_), as a transition metal oxide with a band gap of 2.0–2.2 eV, has been explored extensively. Because of its various forms, high photocurrent density and good thermodynamic stability, it was used widely in photocatalysis research^29^. A lot of studies have been conducted to form a heterojunction between Fe_2_O_3_ and g-C_3_N_4_^30–32^, and the results confirmed that when the heterojunction was formed, the electrons of Fe_2_O_3_ can be transferred to the conduction band of g-C_3_N_4_ to consume holes under light conditions, thus reducing the electron-holes recombination in g-C_3_N_4_ to improve the catalytic activity of composite catalysts. MoS_2_ is a transition metal sulfide with a band gap of about 1.9 eV, has a wide spectral absorption range and can provide migration channels for carriers^33^. Previous studies have proved that MoS_2_ is an effective cocatalyst that can significantly improve the photocatalytic activity of g-C_3_N_4_^34^. However, the photocatalytic hydrogen production rate of the MoS_2_/g-C_3_N_4_ composite catalyst is still relatively low.

At present, there were many works on the composite catalysts of Fe_2_O_3_/g-C_3_N_4_^30,31^and MoS_2_/g-C_3_N_4_^33,35^, but the combination of binary composite catalysts still has the problem of low catalytic rate, so in this research, we combined g-C_3_N_4_ with Fe_2_O_3_ and MoS_2_ to synthesize a new ternary composite catalyst by hydrothermal method to further improve the rate of catalysis. After exploring the addition ratio of Fe_2_O_3_ and MoS_2_, the best ratio of composite catalyst was determined. The increase in the specific surface area of the composite catalyst provided more reaction sites for the photocatalytic reaction. The addition of Fe_2_O_3_ and MoS_2_ accelerated the migration of carriers and the separation of photogenerated electrons and holes. The hydrogen production rate of the composite catalyst was 7.82 mmol g^−1^ h^−1^, which was 5 times higher than the basic catalyst g-C_3_N_4_. Besides, this article also explored the photocatalytic mechanism and stability of the composite catalyst.

## Experimental method

### Synthesis of photocatalysts

Melamine (99%), (NH_4_)_2_CO_3_ and Fe(NO_3_)_3_.9H_2_O(> 98%) were purchased from Sinopharm Chemical Reagent Co., Ltd. Na_2_MoO_4_·2H_2_O was purchased from Jiu ding chemical Technology Co., Ltd. L-cysteine and Chloroplatinic acid hexahydrate (H_2_PtCl_6_·6H_2_O) were purchased from Macklin Biochemical Co., Ltd. Lactic acid was purchased from Ling feng Chemical Reagent Co., Ltd. All reagents are not treated separately.

Preparation of g-C_3_N_4_: In general, melamine was used as a precursor in a muffle furnace to synthesize g-C_3_N_4_ in an air atmosphere. 5 g melamine was put in a crucible with a lid and calcined at 550 °C for 4 h, with a heating rate of 2.5 °C/min. After the calcination was completed, it was cooled and ground the obtained lump g-C_3_N_4_ to obtain light yellow powder, denoted as CN.

Preparation of MoS_2_: Typically, 1.21 g Na_2_MoO_4_·2H_2_O and 1.56 g L-cysteine were dissolved in 50 mL deionized water, stirred for 30 min to fully dissolve the solid. Then the solution was diverted to an autoclave and reacted at 220 °C for 24 h. The sample was separated by centrifugation and then washed three times with deionized water. The obtained black solid was dried in an oven at 65 °C and named MoS_2_.

Preparation of MoS_2_/Fe_2_O_3_/g-C_3_N_4_: MoS_2_/Fe_2_O_3_/g-C_3_N_4_ composite catalyst was prepared by hydrothermal method. Typically, 500 mg CN and 5 mg MoS_2_ were dissolved in 50 mL deionized water, 25 mg Fe(NO_3_)_3_·9H_2_O solution and 10 mL (NH_4_)_2_CO_3_ (0.5 M) solution were added in it, then the above suspension was sonicated for 30 min and transferred to an autoclave before it was hydrothermally heated at 180 °C for 12 h. When the autoclave cooled down to room temperature, the material thus obtained was separated by centrifugation and then washed three times with deionized water and dried in an oven at 65℃. Then the obtained material was ground, and the obtained light red powder was named 1%MoS_2_/Fe_2_O_3_/CN (1% refers to the mass ratio of MoS_2_ to CN). The other samples with 0.5%, 2% and 4% ratios of MoS_2_ were prepared. Fe_2_O_3_ and Fe_2_O_3_/CN were prepared according to the above procedures. The addition ratios of Fe(NO_3_)_3_·9H_2_O to CN were 3wt%, 5wt%, 10wt%, 15wt% respectively. Different ratios of Fe_2_O_3_/CN were named 3%Fe_2_O_3_/CN, 5%Fe_2_O_3_/CN, 10% Fe_2_O_3_/CN and 15% Fe_2_O_3_/CN.

### Characterization

The structures of samples were determined on an X-ray diffractometer (XRD, 18KW/D/max2550VB/PC), the scanning range from 10°-80°. The FT-IR spectra were obtained in the range of 7800–350 cm^−1^. A field electron emission microscope (SEM, Gemini SEM 500) and transmission electron microscope (TEM, JEM-1400) were applied to characterize the structures of the catalysts. The dates of X-ray photoelectron spectroscopy (XPS) were obtained on ESCALAB 250 (Thermo, USA) equipped with an Al Kα radiation source. The specific surface area and pore size distribution were measured through nitrogen adsorption–desorption (Angstroms/3Flex, USA) at 77 K. The UV–Vis absorption spectra ranging from 200 to 800 nm were determined by a Scan UV–Vis spectrophotometer (America, Lambda 950). The photoluminescence (PL) spectra were acquired by Fluorescence Spectrofluorometer. An electrochemical station was used to measure photocurrent responses and electrochemical impedance spectra.

### Photocatalytic hydrogen production

The visible-light-induced H_2_ release was measured in a closed photocatalytic online detection cycle system (CEL-SPH2N, Au Light, Beijing). A 300 W (λ > 400 nm) xenon lamp was selected as the visible light source. 0.05 g catalyst was stirred and dispersed in a glass container containing 20 mL sacrificial agent-Lactic acid and 80 mL distilled water. 2wt% Pt co-catalyst was photodeposited on the as-synthesized catalyst by using H_2_PtCl_6_·6H_2_O as a precursor. The photocatalytic reaction system was evacuated before starting the reaction and the temperature of the reaction solution was maintained at 6 °C by the reaction of cooling water during the photocatalytic reaction. Using nitrogen as the carrier gas, the produced gas was analyzed by a gas chromatograph (GC7900) equipped with a thermal conductivity detector (TCD).

## Results and discussion

### Catalyst characterizations

**Figure 1:**
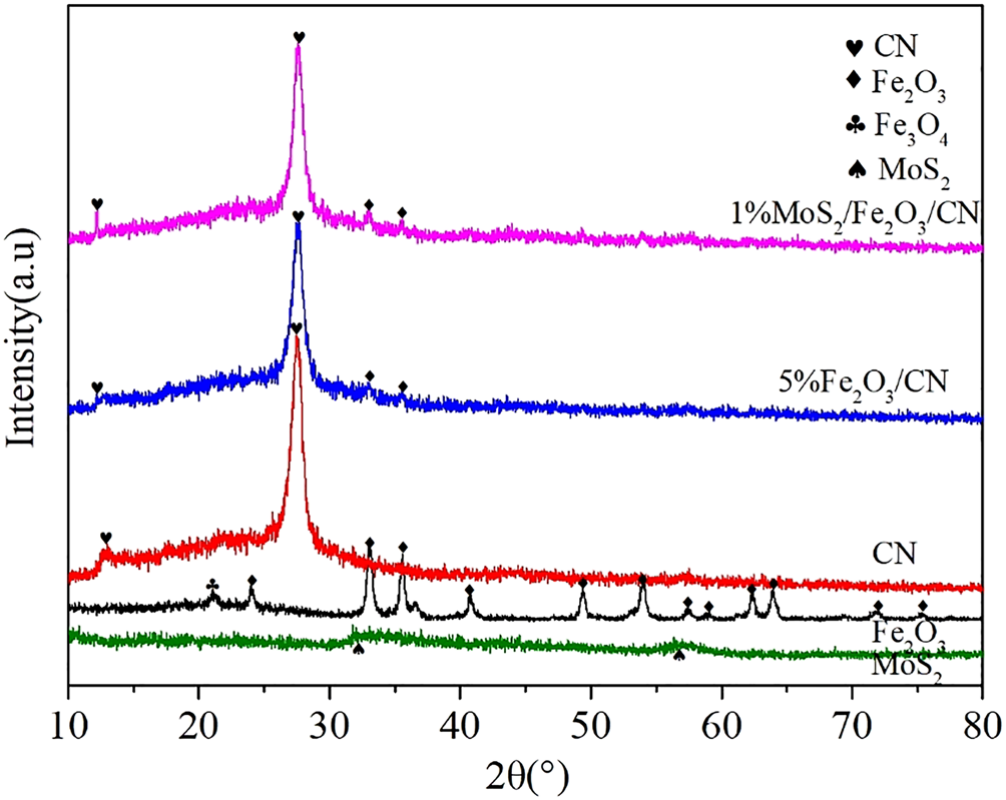
XRD pattern of CN, Fe_2_O_3_, MoS_2_, 5%Fe_2_O_3_/CN and 1%MoS_2_/Fe_2_O_3_/CN.

The XRD results of CN, Fe_2_O_3_, MoS_2_, Fe_2_O_3_/CN and MoS_2_/Fe_2_O_3_/CN were depicted in Fig. 1. There were two characteristic peaks of CN、Fe_2_O_3_/CN and MoS_2_/Fe_2_O_3_/CN at 12.78° and 27.48°, which was consistent with the (100) and (002) planes of CN (JCPDS No.87–1526)^36^. Fe_2_O_3_/CN and MoS_2_/Fe_2_O_3_/CN composite catalyst showed the diffraction peaks of Fe_2_O_3_, but only two obvious characteristic peaks at 33.1°and 35.6° appeared in the composite catalyst because of the low content of Fe_2_O_3_, which corresponding to the (104) and (110) planes respectively^37^. Own to the weak crystallinity and ultra-thin layered structure of MoS_2_, the typical diffraction peaks of MoS_2_ were almost hardly observed in the MoS_2_/Fe_2_O_3_/CN composite catalyst^38^.

**Figure 2:**
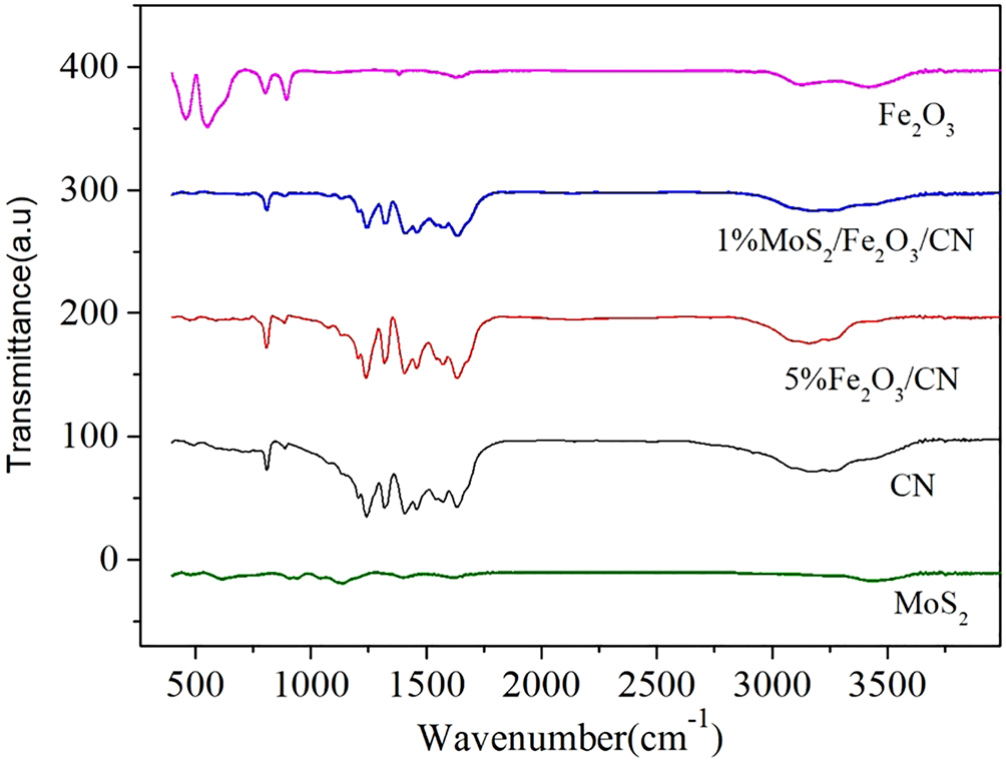
The FT-IR spectra of CN, Fe_2_O_3_, MoS_2_, 5%Fe_2_O_3_/CN and 1%MoS_2_/Fe_2_O_3_/CN.

Figure 2 described the FT-IR spectra of CN, Fe_2_O_3_, MoS_2_, 5%Fe_2_O_3_/CN and 1%MoS_2_/Fe_2_O_3_/CN photocatalysts. The photocatalysts of CN, 5%Fe_2_O_3_/CN and 1%MoS_2_/Fe_2_O_3_/CN all had the typical peaks of g-C_3_N_4_ in the FT-IR spectra, corresponding to the results of XRD. The characterize peaks at 1240–1650 cm^−1^ were originated from the typical C–N and C=N stretching of heterocyclic compounds. The strong peak at 810 cm^−1^ represented the triazine unit in CN^36^. The broad peak ranging from 3000 to 3300 cm^−1^ can be attributed to the N–H bond stretching of the unreacted amino group. Because of the low content of Fe_2_O_3_ and MoS_2_, the characteristic peaks of Fe–O and Mo-S were hardly observed in the samples of 5%Fe_2_O_3_/CN and 1%MoS_2_/5%Fe_2_O_3_/CN, which same as the results in XRD.

**Figure 3:**
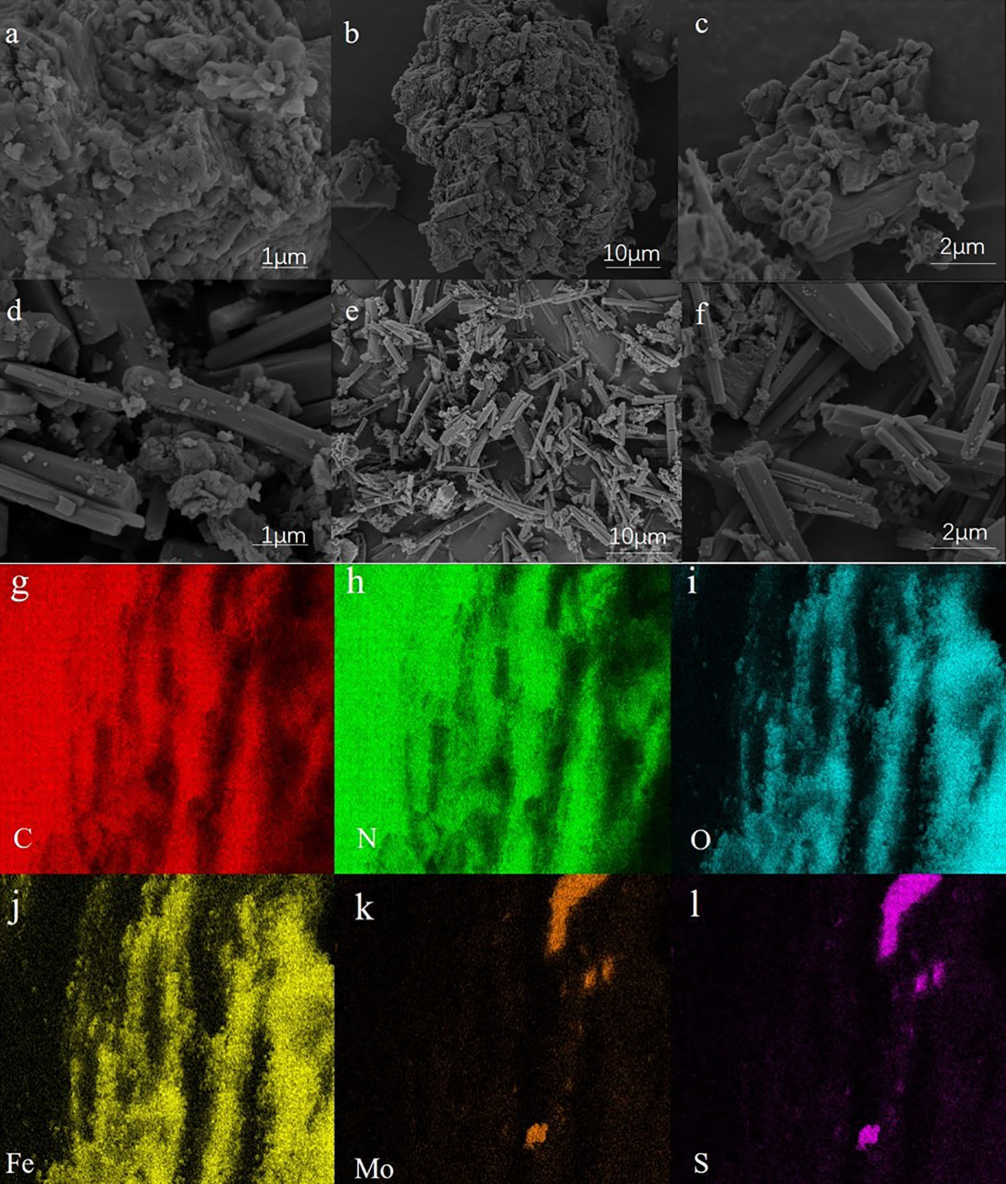
SEM images of CN: (**a**) 40,000×, (**b**) 5000×, (**c**) 20,000×, MoS_2_/Fe_2_O_3_/CN (**d**). 40,000×, (**e**) 5000×, (**f**) 20,000×, and the corresponding elements mapping of MoS_2_/Fe_2_O_3_/CN.

The specific morphology of the catalyst was of great significance to the study of its properties. The microscopic morphology of CN and MoS_2_/Fe_2_O_3_/CN was acquired by SEM and the results were illustrated in Fig. 3. Figure 3a–c displayed the morphology and structure of CN at different multiples. CN was an irregular block structure, which was consistent with the literature report^39,40^. And the block structure of CN led to its small specific surface area, which was also a significant reason for its low catalytic efficiency. Figure 3d–f showed the morphology and structure of MoS_2_/Fe_2_O_3_/CN at different multiples. The structure of the composite catalyst changed during the formation process, from the block structure to the rod structure, which was mainly attributed to the hydrothermal reaction. There were some small particles on the rod-shaped structure, but it was hard to judge whether there were traces of Fe_2_O_3_ and MoS_2_. And it was difficult to clearly distinguish them in the SEM image because of the low content of Fe_2_O_3_ and MoS_2_ in the composite catalyst. And the elemental composition of MoS_2_/Fe_2_O_3_/CN was determined by energy- dispersive X-ray spectroscopy (EDS) mapping from Fig. 3g–o. From the results, elements of C, N, Fe, O, Mo and S can be captured.

**Figure 4:**
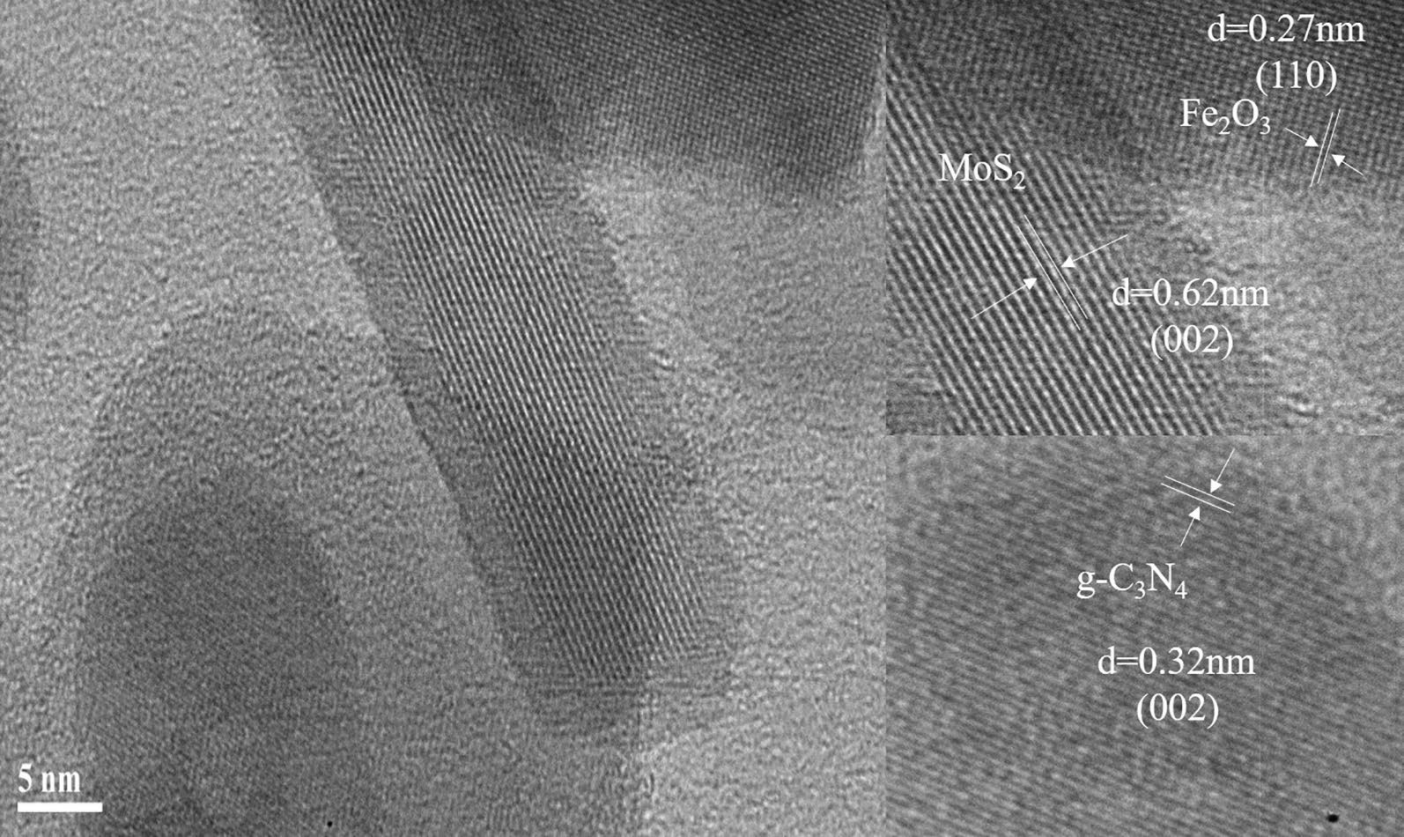
The HRTEM images of MoS_2_/Fe_2_O_3_/CN composite catalyst.

The detailed structure information of the MoS_2_/Fe_2_O_3_/CN composite catalyst were demonstrated in HRTEM, and the results were depicted in Fig. 4. Fe_2_O_3_ and MoS_2_ form a composite catalyst with CN in the hydrothermal process. Three lattice fringes were detected in the figure of HRTEM, and the corresponding lattice spacing were 0.62 nm, 0.32 nm and 0.27 nm, which were attributed to the (002) plane of MoS_2_, the (002) plane of CN and the (110) plane of Fe_2_O_3_^31,41,42^. The positional combination of the three materials provided evidence for the charge transfer pathway of the photocatalytic mechanism.

**Figure 5:**
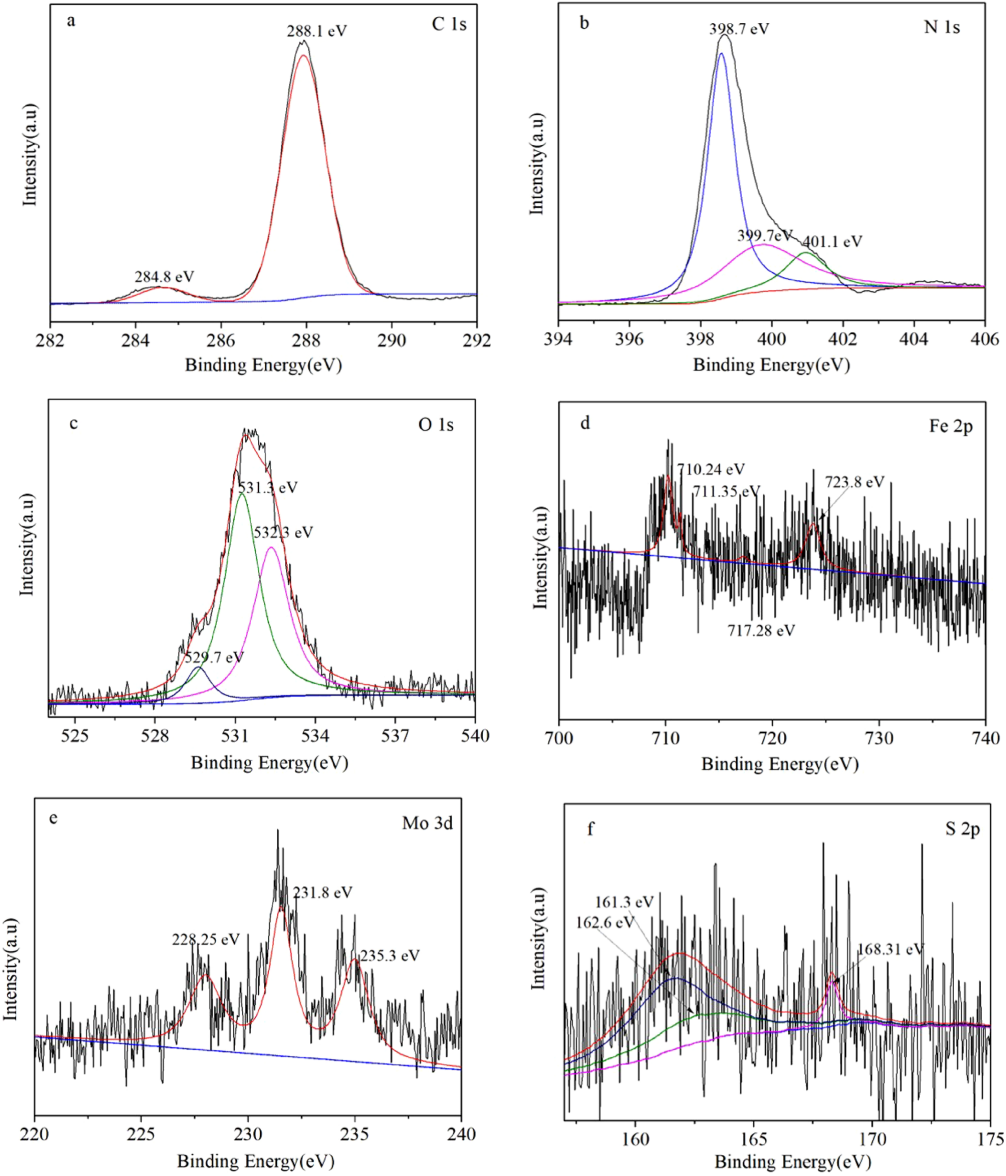
XPS spectra of 1%MoS_2_/Fe_2_O_3_/CN, (**a**) C 1s spectrum, (**b**) N 1s spectrum, (**c**) O 1s spectrum, (**d**) Fe 2p spectrum, (**e**) Mo 3d spectrum, (**f**) S 2p spectrum.

Because of the small content of Fe_2_O_3_ and MoS_2_ in the composite catalyst, it was hard to find information of them in SEM, XRD and FT-IR, but the successful combination of Fe_2_O_3_, MoS_2_ and CN were proved in EDS and HRTEM results. In order to further explore the characteristics of MoS_2_/Fe_2_O_3_/CN, the element composition and chemical state of the MoS_2_/Fe_2_O_3_/CN composite catalyst were acquired through XPS. The XPS spectrums of various elements were demonstrated in Fig. 5, and some vestige of Fe_2_O_3_ and MoS_2_ were found in the XPS spectrums. There were two peaks at 284.8 eV and 288.2 eV in C 1s spectrum, which consistent with the bond of C–C and N=C–N respectively^43^. Figure 5b illustrated the N 1s spectrum of the composite catalyst, it contained three peaks at 398.7 eV, 399.7 eV and 401.1 eV. The peak at 398.7 eV was caused by sp^2−^ heterozygosity N in C=N–C. The peak at 399.7 eV corresponded to the sp^3−^ hybridization of N in N–(C)_3_, while the peak at 401.1 eV was arose from N–C-H in the amino group. The O 1s spectrum appeared three characteristic peaks at 529.7 eV, 531.3 eV and 532.3 eV, which can be ascribed to the O^2−^ in Fe_2_O_3_, hydroxyl in the surface and the adsorption of H_2_O^37,44^. The Fe 2p spectrum had four peaks at 710.24 eV, 711.35 eV, 717.28 eV and 723.8 eV. 711.35 eV and 723.8 eV corresponded to the binding energies of Fe 2p_5/2_ and Fe 2p_1/2_, which were consistent with Fe^3+^ in Fe_2_O_3_. The two satellite peaks at 710.24 eV, 717.28 eV were caused by the charge migration of Fe^3+^^44–46^. The two peaks at 228 eV, 231.8 eV in Mo 2p spectrum can be attributed to Mo 3d_5/2_ and 3d_3/2_, indicating that the existence of Mo^4+^^33^. The peak at 235.3 eV was corresponded to the Mo–O bond of Mo 6p, which was formed by the combination of Mo atoms and O in Fe_2_O_3_^47^. The XPS spectrum of S 2p was displayed in Fig. 5d, the characteristic peaks at 161.3 eV and 162.6 eV have corresponded to S 2p_3/2_ and S 2p_1/2_ of S^2−^. The peak at 168.31 eV belonged to the edge unsaturated S atom of the ultra-thin layer, which was considered to be the active site for hydrogen production and was the unique flake modification advantage of MoS_2_^47^. The unique advantage of MoS_2_ lied in the presence of edge unsaturated S atom, which provided more reaction site to improve the hydrogen production. The binding energy corresponding to the unsaturated S atom was 168.31 eV.

**Figure 6:**
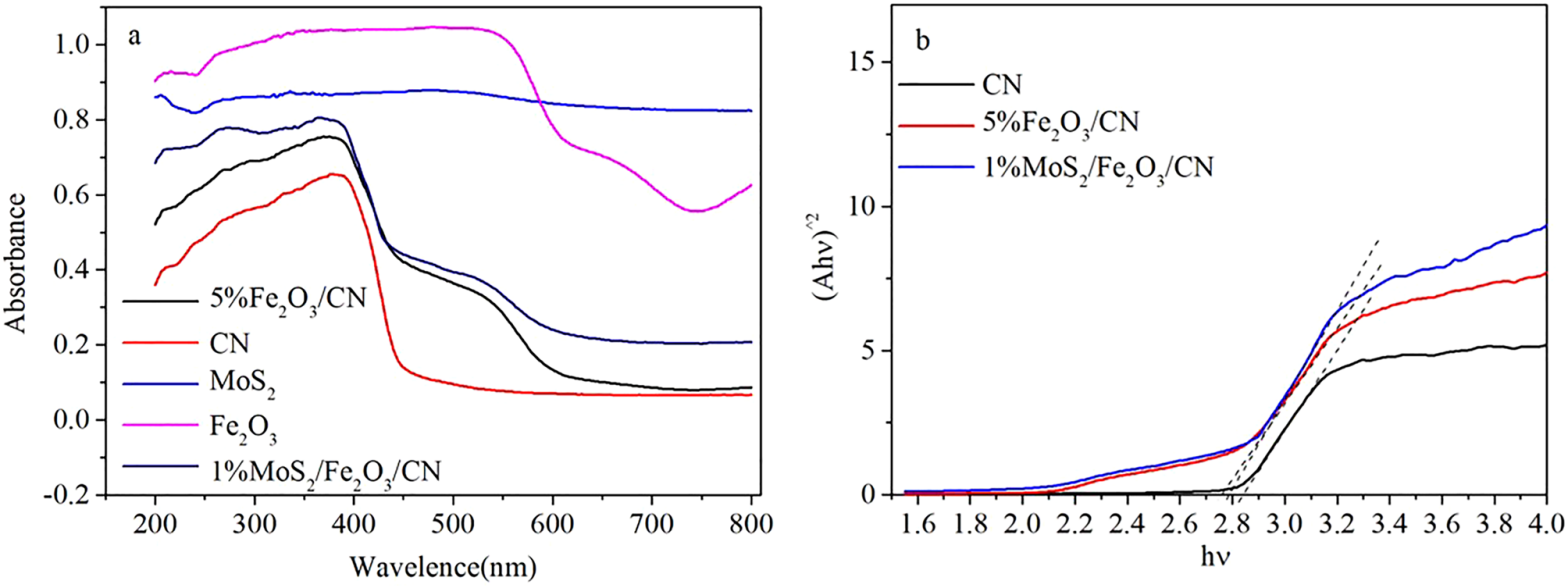
(**a**) UV–Vis absorption spectrum of photocatalysts (**b**) band gap diagram of photocatalysts.

Figure 6 depicted the UV–Vis diffuse reflectance spectra of CN, Fe_2_O_3_, MoS_2_, Fe_2_O_3_/CN and MoS_2_/Fe_2_O_3_/CN composite catalysts. In the range of 300–450 nm, the catalyst of CN had higher absorption and appeared an absorption edge at about 456 nm. The UV absorption spectrum of the Fe_2_O_3_/CN composite catalyst was similar to the main absorption edge of CN. The composite catalyst had two absorption band edges, which were consistent with the UV absorption spectrum of Fe_2_O_3_. The composite catalyst exhibited stronger absorption, especially under visible light, and the absorption curve shifted significantly to longer wavelengths. After MoS_2_ loaded to Fe_2_O_3_/CN composite catalyst, the absorption of visible light by the composite catalyst had been further improved. The band gap value of the photocatalyst was determined by the following formula.

$$\alpha h\nu =A{(hv-{E}_{g})}^{n/2}$$

where α, hν, and E_g_ refers to absorption coefficient, photon energy and band gap, respectively. A is a constant, n = 1 means a direct band gap while n = 4 refers to an indirect band gap^48^. According to the equation, the band gaps of CN, Fe_2_O_3_/CN and MoS_2_/Fe_2_O_3_/CN can be roughly estimated, the results were shown in Fig. 6b. According to the formula, the band gap of CN was predicted to be 2.84 eV. Besides, the band gap values of the composite catalysts 5%Fe_2_O_3_/CN and 1%MoS_2_/Fe_2_O_3_/CN were 2.76 eV and 2.79 eV according to the prediction. The band gap of the synthesized composite catalysts were reduced, which were beneficial to the catalyst to further absorb sunlight and improve the photocatalytic performance.

**Figure 7:**
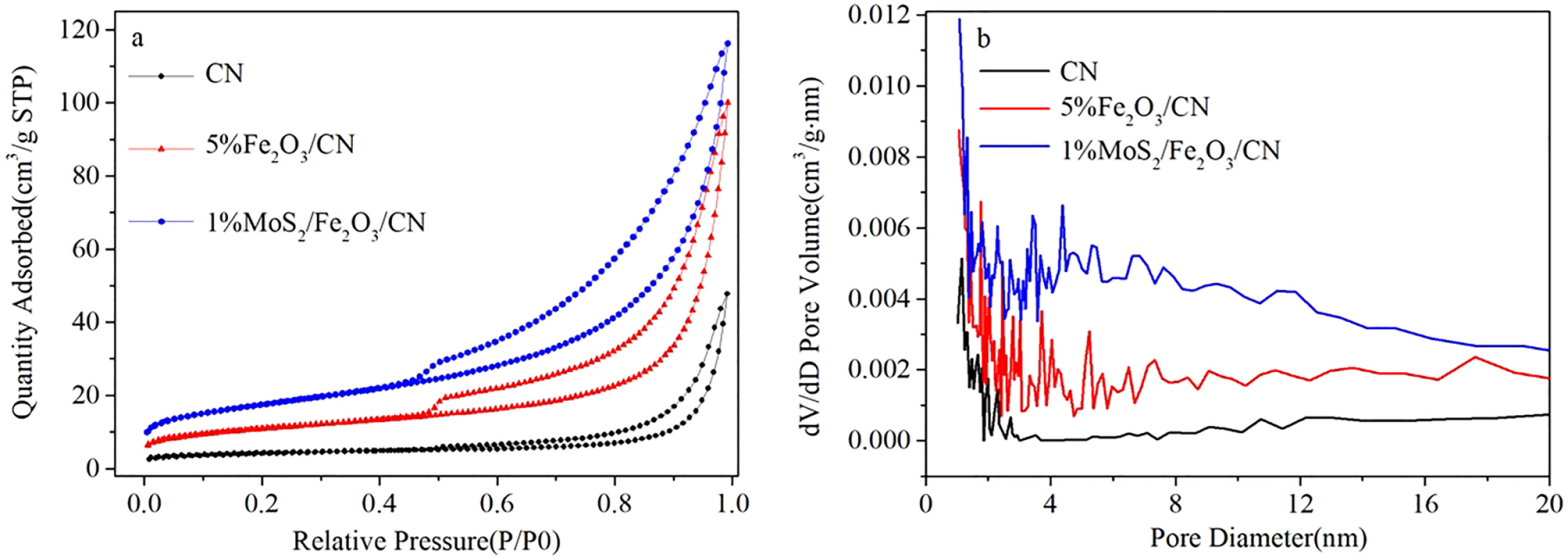
N_2_ adsorption–desorption isotherms and pore size distributions of CN, 5%Fe_2_O_3_/CN, and 1% MoS_2_/Fe_2_O_3_/CN.

Table 1 described the specific surface area of CN, 5%Fe_2_O_3_/CN and 1%MoS_2_/Fe_2_O_3_/CN. As illustrated, the specific surface area of 1%MoS_2_/Fe_2_O_3_/CN was 61.76 m^2^/g, which was the highest among all catalysts, consistent with the results of the highest H_2_ production rate discussed below. The specific surface area of the composite catalyst was increased mainly due to the change of the composite catalyst in structure, from the original block structure to a rod-like structure during the hydrothermal reaction process. This structure can provide more sites for the catalytic reactions. And the addition of MoS_2_ increased the number of micropores in the composite catalyst, thereby further increasing the specific surface area of the composite catalyst. As we all know that the specific surface area was one of the main reasons influencing the performance of the photocatalyst. High specific surface area can provide more active sites for photocatalytic reaction and the existence of the pore structure provide more carrier charge transfer channels, which helped to improve the photocatalytic activity. The nitrogen adsorption–desorption isotherms of CN, 5%Fe_2_O_3_/CN and 1%MoS_2_/Fe_2_O_3_/CN were demonstrated in Fig. 7. According to the classification of IUPAC, the adsorption–desorption isotherm of the photocatalysts of CN, 5%Fe_2_O_3_/CN and 1%MoS_2_/Fe_2_O_3_/CN was type IV, and the types of hysteresis loop were H3. As depicted, when the relative pressure was in the range of 0.8–1.0, the adsorption–desorption isotherm appears higher absorption, which meant that the catalyst had a porous structure.

**Table 1:** The specific surface areas of CN, 5%Fe_2_O_3_/CN and 1%MoS_2_/Fe_2_O_3_/CN.

Sample	CN	5%Fe_2_O_3_/CN	1%MoS_2_/Fe_2_O_3_/CN
S_BET_ (m^2^/g)	14.55	38.36	61.76

**Figure 8:**
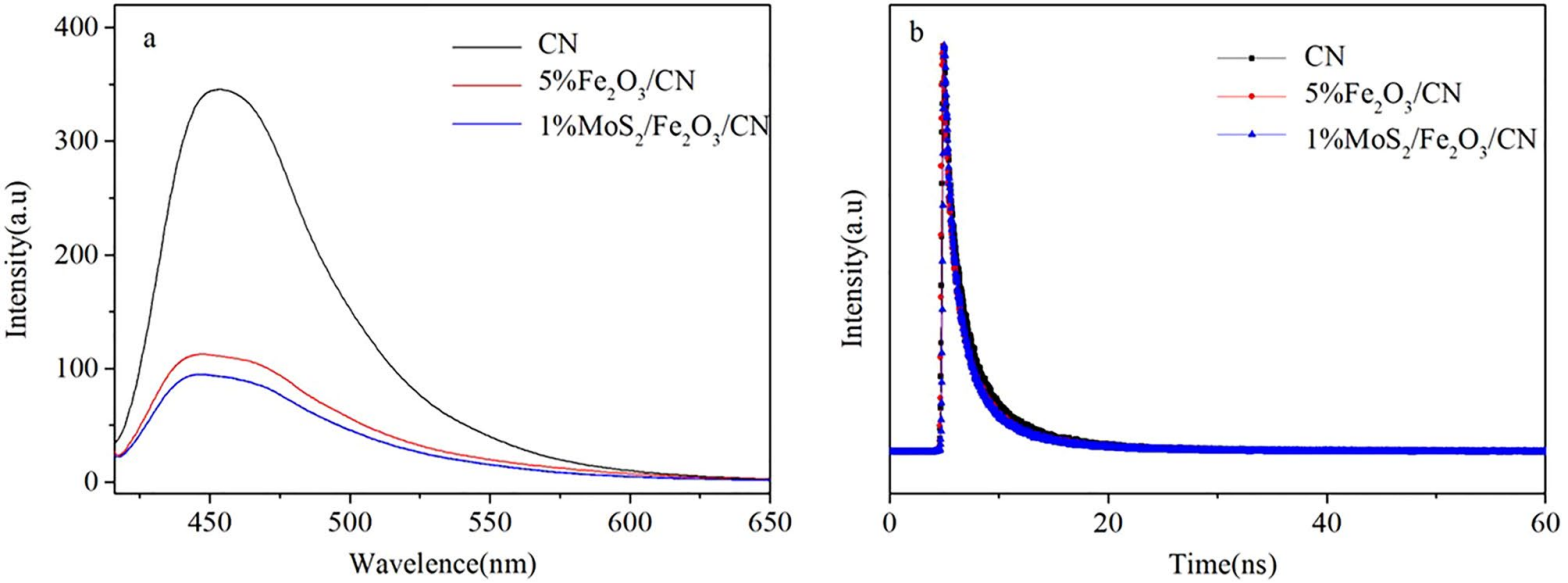
The PL spectrum (**a**) and fluorescence decay curves (**b**) of CN, 5%Fe_2_O_3_/CN and 1%MoS_2_/Fe_2_O_3_/CN.

To compare the carrier separation efficiency of different photocatalysts, CN, 5%Fe_2_O_3_/CN and 1%MoS_2_/Fe_2_O_3_/CN were characterized by photoluminescence spectroscopy and time-resolved PL spectra, the results were described in Fig. 8a, b. The PL spectrum can display the photogenerated electron–hole separation ability of the prepared composite catalyst. Generally, the stronger the spectrum intensity of the PL, the lower the separation efficiency of the photocatalyst carriers. The PL spectrum of pure CN had very high fluorescence intensity in the range of 420–550 nm, which was about 4 times higher than the composite catalyst MoS_2_/Fe_2_O_3_/CN. It meant that the pure CN had weaker carrier mobility than the composite catalyst, and photo-generated electron–hole was more likely to occur complex. The fluorescence intensity of the MoS_2_/Fe_2_O_3_/CN composite catalyst was the weakest, indicating it had the highest carrier separation efficiency and lowest probability of photo-generated electron–hole recombination among all photocatalysts. Time-resolved PL spectroscopy further demonstrates the characteristics of charge carrier lifetime. The average lifetime of MoS_2_/Fe_2_O_3_/CN was 4.954 ns, which shorter than CN (5.717 ns) and Fe_2_O_3_/CN (5.538 ns), it meant MoS_2_/Fe_2_O_3_/CN can effectively inhibit the recombination of electron and hole. The PL and time-resolved PL spectra results were consistent with the conclusion of the hydrogen production test.

**Figure 9:**
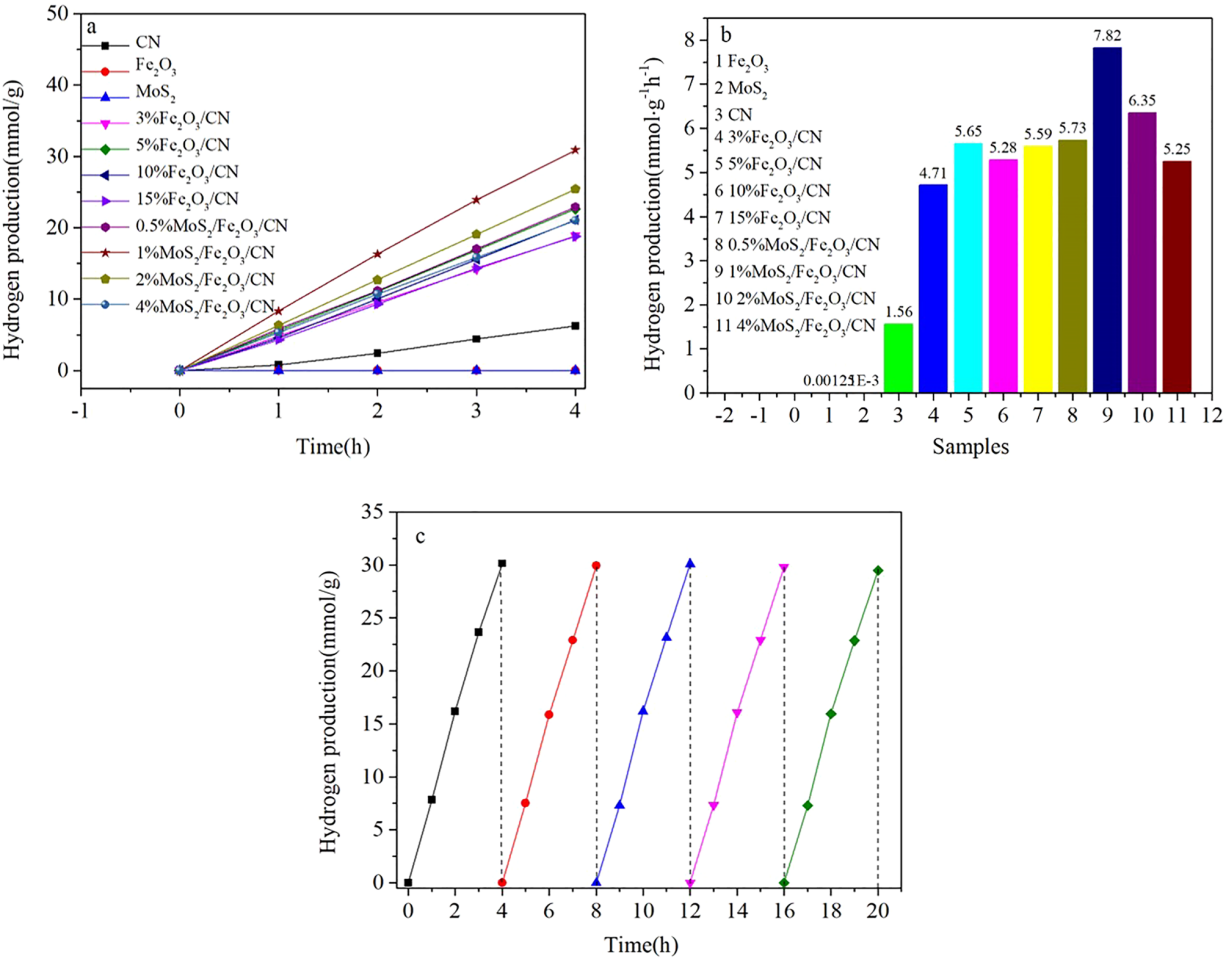
(**a**) The Hydrogen accumulation of different samples, (**b**) the Hydrogen producton rate of different samples, (**c**) the hydrogen production cycle of 1%MoS_2_/Fe_2_O_3_/CN composite catalyst.

Under visible light condition, the hydrogen production rate of MoS_2_/Fe_2_O_3_/CN composite catalyst was shown in Fig. 9, including the control groups. The hydrogen production rate of pure CN was 1.56 mmol g^−1^ h^−1^, while the rates of Fe_2_O_3_ and MoS_2_ were relatively low, which can be almost ignored. Fe_2_O_3_/CN composite catalyst demonstrated enhanced hydrogen release performance and the 5%Fe_2_O_3_/CN photocatalyst showed the best hydrogen production rate, which was 5.65 mmol g^−1^ h^−1^. After loading MoS_2_ on the Fe_2_O_3_/CN composite catalyst, the photocatalytic hydrogen production rate of the composite catalyst was further improved. The highest hydrogen production rate of 1%MoS_2_/Fe_2_O_3_/CN composite catalyst was 7.82 mmol g^−1^ h^−1^, which was about 5 times higher than that of pure CN (1.56 mmol g^−1^ h^−1^) and 1.38 times higher than 5%Fe_2_O_3_/CN. The reusability and stability of the catalyst were considered to be important practical evaluation and application parameters. In this paper, using MoS_2_/Fe_2_O_3_/CN as the photocatalyst, five consecutive cycles of experiments were conducted to explore the stability of hydrogen evolution. As displayed in Fig. 9c, the hydrogen generation rate was still stable after five cycles, indicating the good stability and sustainable utilization of the photocatalyst.

### Electrochemical characterization

**Figure 10:**
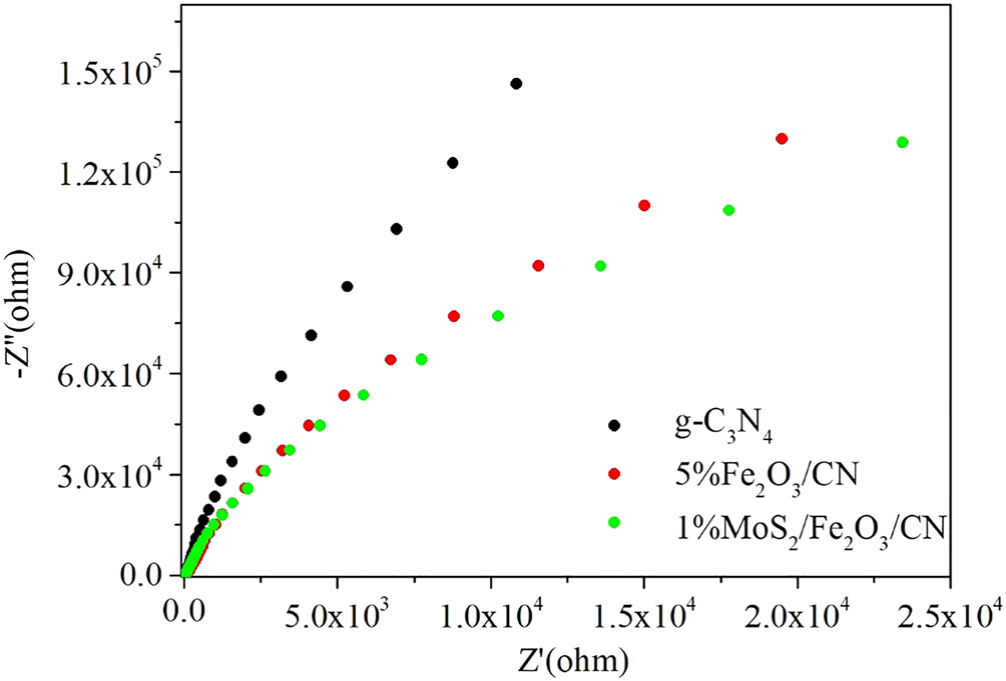
Electrochemical impedance spectra of samples.

An electrochemical impedance spectroscopy test was carried out to evaluate the charge transfer capability of the photocatalyst, and the results were demonstrated in Fig. 10. As described in Fig. 10, the resistance of pure CN was relatively large, which hindered its charge transfer. After being combined with 5% Fe_2_O_3_, the resistance of the composite catalyst was greatly decreased. When the catalyst was further combined with MoS_2_, the charge transfer resistance of the composite catalyst was further decreased. It was known that when the resistance of electrochemical impedance was smaller, the resistance of charge transfer was lower, the photocatalytic activity was better. The results of PL demonstrated that the charge transferability of the MoS_2_/Fe_2_O_3_/CN composite catalyst was improved, thus the photocatalytic performance was improved.

**Figure 11:**
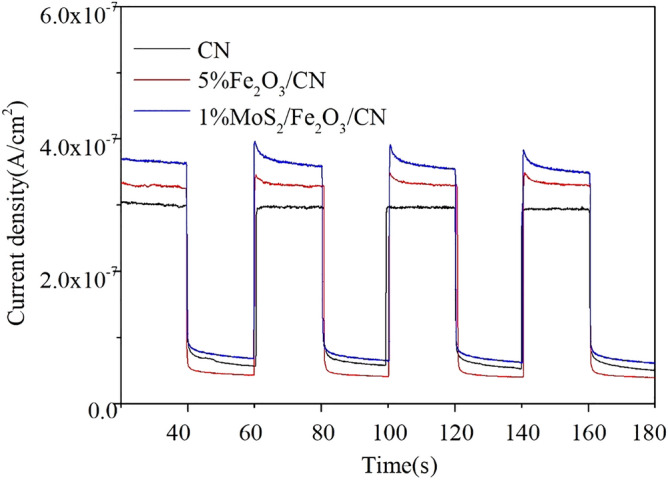
Transient photocurrent responses of photocatalysts.

The test of transient photocurrent response was an effective way to evaluate the carrier mobility of photocatalysts. And the photocurrent responses diagrams of CN, 5%Fe_2_O_3_/CN and 1%MoS_2_/Fe_2_O_3_/CN photocatalysts were shown in Fig. 11. The photocurrent responses current of the composite catalyst 1%MoS_2_/Fe_2_O_3_/CN was significantly higher than the catalysts of 5%Fe_2_O_3_/CN and CN, which implied that its electron–hole separation efficiency was the highest among the samples. And the results also illustrated that the photocatalytic activity of the composite catalyst was the best, which was consistent with the results of PL and EIS.

### Photocatalytic mechanism

**Figure 12:**
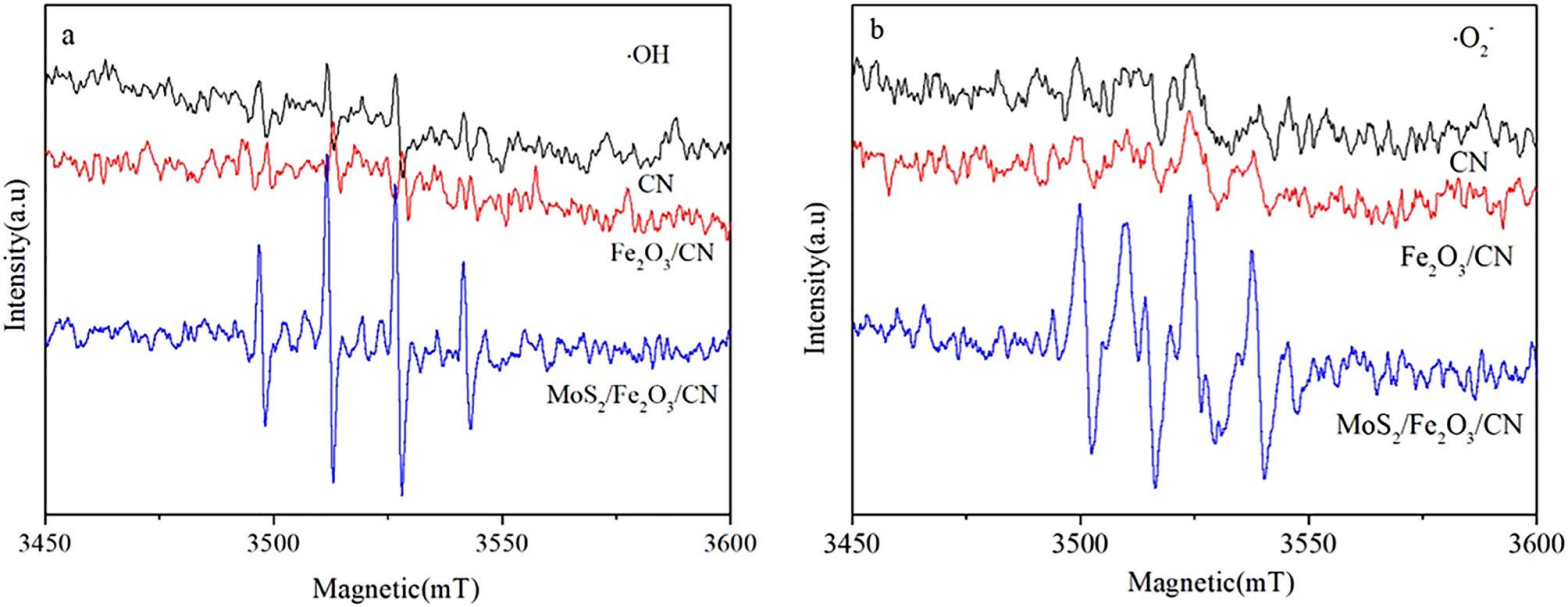
EPR spectra of CN, Fe_2_O_3_/CN and MoS_2_/Fe_2_O_3_/CN in different system under visible light. (**a**) DMPO − ·OH in water, (**b**) DMPO − ·O_2_^−^ in methanol in the system.

**Figure 13:**
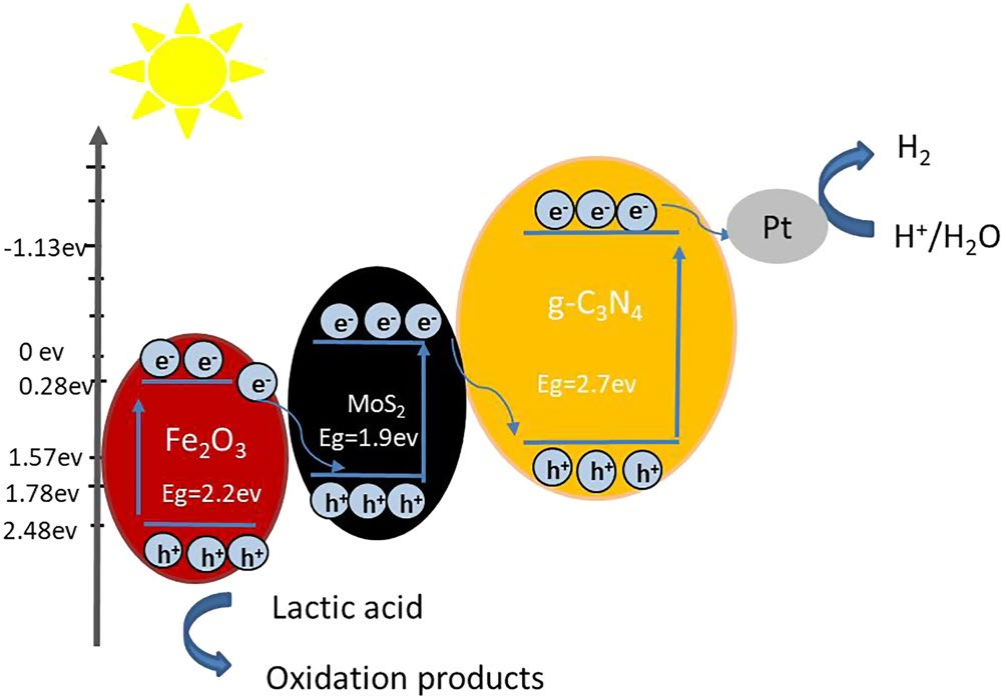
The photocatalytic mechanism of 1%MoS_2_/Fe_2_O_3_/CN composite catalyst.

In order to explore the process mechanism of photocatalytic hydrogen production, the EPR characterization technology was used to detect the free radicals in the photocatalytic process. The test results were shown in Fig. 12 and the EPR signals of ·OH and ·O_2_^−^ were shown in Fig. 12a, b, respectively. The results demonstrated that signals of ·OH and ·O_2_^−^ were existed in the system of MoS_2_/Fe_2_O_3_/CN and the peak intensity of MoS_2_/Fe_2_O_3_/CN was the highest, which indicated that the catalytic mechanism of the ternary composite catalyst can be represented by Z-scheme. Based on the EPR and HRTEM results, the possible photocatalytic mechanism of the MoS_2_/Fe_2_O_3_/CN composite catalyst was illustrated in Fig. 13. The band gap structures of g-C_3_N_4_, Fe_2_O_3_ and MoS_2_ were determined by previous studies^49,50^. Under the condition of light, electrons were generated in the valence bands of CN、Fe_2_O_3_ and MoS_2_, the electrons transferred from the valence band to the conduction band, and holes were generated in the valence band. It was known from the literature that if the reaction was based on the traditional heterojunction electron transfer mechanism, the active materials ·OH and ·O_2_^−^ cannot be produced due to the potential of CN、Fe_2_O_3_ and MoS_2_^46^, which was inconsistent with the EPR test results in Fig. 12. Therefore, based on the previously reported literature, in this paper, we explained the photocatalytic performance of the composite catalyst through the Z-type heterojunction mechanism. In this system, the electrons in the Fe_2_O_3_ conduction band were transferred to the valence band of CN under the action of the intermediary MoS_2_, which consumed the holes and reduced the photo-generated electron–hole recombination in CN. The sacrificial agent lactic acid was oxidized on the valence band of Fe_2_O_3_, consuming holes and accelerating the migration of carriers. Under the action of the co-catalyst MoS_2_ and Pt, the active sites of CN can effectively produce hydrogen.

## Conclusions

In conclusion, the ternary composite catalyst MoS_2_/Fe_2_O_3_/CN was successfully prepared through the hydrothermal method, and a series of characterizations confirmed that the composite catalyst has the superior photocatalytic performance among the samples. The various characterization results demonstrated that with the addition of Fe_2_O_3_ and MoS_2_, the ternary composite catalyst MoS_2_/Fe_2_O_3_/CN had a larger specific surface area, stronger visible light absorption capacity, higher carrier migration efficiency, and lower photogenerated electron–hole recombination rate. And MoS_2_/Fe_2_O_3_/CN showed higher hydrogen production activity, reaching 7.82 mmol g^−1^ h^−1^, which was about 5 times higher than the basic catalyst CN. The Z-scheme photocatalytic process was testified by EPR analysis and the research on the composite catalyst MoS_2_/Fe_2_O_3_/CN provided some useful reference information for the development of other ternary composite catalysts.

## References

[1] Wang Z, Huang X, Wang X (2019). Recent progresses in the design of BiVO_4_-based photocatalysts for efficient solar water splitting. Catal. Today.

[2] Guo H, Chen M, Zhong Q, Wang Y, Ma W, Ding J (2019). Synthesis of Z-scheme α-Fe_2_O_3_/g-C_3_N_4_ composite with enhanced visible-light photocatalytic reduction of CO_2_ to CH_3_OH. J. CO2 Util..

[3] Dong Z, Wu Y, Thirugnanam N, Li G (2018). Double Z-scheme ZnO/ZnS/ g-C_3_N_4_ ternary structure for efficient photocatalytic H_2_ production. Appl. Surf. Sci..

[4] Kahng S, Yoo H, Kim JH (2020). Recent advances in earth-abundant photocatalyst materials for solar H_2_ production. Adv. Powder Technol..

[5] Zhang X, Li L, Zhou Q, Liang X, Liu D (2019). Facile synthesis of novel gully-like double-sized mesoporous structural Sr-doped ZrO_2_–TiO_2_ composites with improved photocatalytic efficiency. J. Solid State Chem..

[6] Bo T, Yuan J, Liu Y, Cao S, Zhou W (2021). Activated edge of single layered TiO_2_ nanoribbons through transition metal doping and strain approaches for hydrogen production. Appl. Surf. Sci..

[7] Ismael M (2020). Enhanced photocatalytic hydrogen production and degradation of organic pollutants from Fe (III) doped TiO_2_ nanoparticles. J. Environ. Chem. Eng..

[8] Lin K, Feng L, Li D, Zhang J, Wang W, Ma B (2021). Improved photocatalytic hydrogen evolution on (Ru/WC)/CdS via modulating the transferring paths of photo-excited electrons. Appl. Catal. B Environ..

[9] Yang H, Yang C, Zhang N, Mo K, Li Q, Lv K (2021). Drastic promotion of the photoreactivity of MOF ultrathin nanosheets towards hydrogen production by deposition with CdS nanorods. Appl. Catal. B Environ..

[10] Zhuge K, Chen Z, Yang Y, Wang J, Shi Y, Li Z (2021). In-suit photodeposition of MoS_2_ onto CdS quantum dots for efficient photocatalytic H_2_ evolution. Appl. Surf. Sci..

[11] Galdámez-Martínez A, Bai Y, Santana G, Sprick RS, Dutt A (2020). Photocatalytic hydrogen production performance of 1-D ZnO nanostructures: role of structural properties. Int. J. Hydrog. Energy.

[12] Haddad M, Belhadi A, Boudjellal L, Trari M (2020). Photocatalytic hydrogen production on the hetero-junction CuO/ZnO. Int. J. Hydrog. Energy.

[13] Patil RP, Mahadik MA, Chae W-S, Choi SH, Jang JS (2021). Self-templated fabrication of 2-D dual nanoarchitecture Zn1-xCdxS porous nanosheet and ZnO nanorod for photoelectrochemical hydrogen production. Appl. Surf. Sci..

[14] Deng P, Hong W, Cheng Z, Zhang L, Hou Y (2020). Facile fabrication of nickel/porous g-C3N4 by using carbon dot as template for enhanced photocatalytic hydrogen production. Int. J. Hydrog. Energy.

[15] Hu Z, Lyu J, Ge M (2020). Role of reactive oxygen species in the photocatalytic degradation of methyl orange and tetracycline by Ag_3_PO_4_ polyhedron modified with g-C_3_N_4_. Mater. Sci. Semicond. Process..

[16] Chen Z, Xia K, She X, Mo Z, Zhao S, Yi J (2018). 1D metallic MoO_2_-C as co-catalyst on 2D g-C_3_N_4_ semiconductor to promote photocatlaytic hydrogen production. Appl. Surf. Sci..

[17] Li Y, Zhou M, Cheng B, Shao Y (2020). Recent advances in g-C_3_N_4_-based heterojunction photocatalysts. J. Mater. Sci. Technol..

[18] Liu G, Qiao X, Gondal MA, Liu Y, Shen K, Xu Q (2018). Comparative study of pure g-C_3_N_4_ and sulfur-doped g-C_3_N_4_ catalyst performance in photo-degradation of persistent pollutant under visible light. J. Nanosci. Nanotechnol..

[19] Lin Q, Li Z, Lin T, Li B, Liao X, Yu H (2020). Controlled preparation of P-doped g-C_3_N_4_ nanosheets for efficient photocatalytic hydrogen production. Chin. J. Chem. Eng..

[20] Liu G, Xue M, Liu Q, Yang H, Zhou Y (2019). Facile synthesis of C-doped hollow spherical g-C3N4 from supramolecular self-assembly for enhanced photoredox water splitting. Int. J. Hydrog. Energy.

[21] Che H, Che G, Zhou P, Liu C, Dong H, Li C (2020). Nitrogen doped carbon ribbons modified g-C_3_N_4_ for markedly enhanced photocatalytic H_2_-production in visible to near-infrared region. Chem. Eng. J..

[22] Sun S, Li J, Song P, Cui J, Yang Q, Zheng X (2020). Facile constructing of isotype g-C_3_N_4_(bulk)/g-C_3_N_4_(nanosheet) heterojunctions through thermal polymerization of single-source glucose-modified melamine: an efficient charge separation system for photocatalytic hydrogen production. Appl. Surf. Sci..

[23] Wang Y, Zhao S, Zhang Y, Fang J, Zhou Y, Yuan S (2018). One-pot synthesis of K-doped g-C_3_N_4_ nanosheets with enhanced photocatalytic hydrogen production under visible-light irradiation. Appl. Surf. Sci..

[24] Dong F, Wu L, Sun Y, Fu M, Wu Z, Lee SC (2011). Efficient synthesis of polymeric g-C_3_N_4_ layered materials as novel efficient visible light driven photocatalysts. J. Mater. Chem..

[25] Hao X, Zhou J, Cui Z, Wang Y, Wang Y, Zou Z (2018). Zn-vacancy mediated electron-hole separation in ZnS/g-C_3_N_4_ heterojunction for efficient visible-light photocatalytic hydrogen production. Appl. Catal. B Environ..

[26] Pan J, Dong Z, Wang B, Jiang Z, Zhao C, Wang J (2019). The enhancement of photocatalytic hydrogen production via Ti3+ self-doping black TiO_2_/g-C_3_N_4_ hollow core-shell nano-heterojunction. Appl. Catal. B Environ..

[27] Zhu Y, Wan T, Wen X, Chu D, Jiang Y (2019). Tunable Type I and II heterojunction of CoOx nanoparticles confined in g-C_3_N_4_ nanotubes for photocatalytic hydrogen production. Appl. Catal. B Environ..

[28] Liu J, Jia Q, Long J, Wang X, Gao Z, Gu Q (2018). Amorphous NiO as co-catalyst for enhanced visible-light-driven hydrogen generation over g-C_3_N_4_ photocatalyst. Appl. Catal. B Environ..

[29] Tong M, Sun D, Zhang R, Liu H, Chen R (2021). Preparation of Si–α-Fe_2_O_3_/CdS composites with enhanced visible-light photocatalytic activity for p-nitrophenol degradation. J. Alloys Compd..

[30] Xiong S, Liu X, Zhu X, Liang G, Jiang Z, Cui B (2021). One-step preparation of well-dispersed spindle-like Fe_2_O_3_ nanoparticles on g-C3N4 as highly efficient photocatalysts. Ecotoxicol. Environ. Saf..

[31] Zhao H, Tian C, Mei J, Yang S, Wong PK (2021). Faster electron injection and higher interface reactivity in g-C_3_N_4_/Fe_2_O_3_ nanohybrid for efficient photo-Fenton-like activity toward antibiotics degradation. Environ. Res..

[32] Zhang R, Bi L, Wang D, Lin Y, Zou X, Xie T (2020). An effective method to understand photo-generated charge transfer processes of Z-scheme Ti/α-Fe_2_O_3_/g-C_3_N_4_ photocatalysts for hydrogen evolution. Catal. Commun..

[33] Zhang X, Zhang R, Niu S, Zheng J, Guo C (2019). Enhanced photo-catalytic performance by effective electron-hole separation for MoS_2_ inlaying in g-C_3_N_4_ hetero-junction. Appl. Surf. Sci..

[34] Sun J, Yang S, Liang Z, Liu X, Qiu P, Cui H (2020). Two-dimensional/one-dimensional molybdenum sulfide (MoS_2_) nanoflake/graphitic carbon nitride (g-C_3_N_4_) hollow nanotube photocatalyst for enhanced photocatalytic hydrogen production activity. J. Colloid Interface Sci..

[35] Zhang Z, Liu C, Dong Z, Dai Y, Xiong G, Liu Y (2020). Synthesis of flower-like MoS_2_/g-C_3_N_4_ nanosheet heterojunctions with enhanced photocatalytic reduction activity of uranium(VI). Appl. Surf. Sci..

[36] Ghane N, Sadrnezhaad SK, Hosseini HSM (2020). Combustion synthesis of g-C_3_N_4_/Fe_2_O_3_ nanocomposite for superior photoelectrochemical catalytic performance. Appl. Surf. Sci..

[37] Bakr AEA, El Rouby WMA, Khan MD, Farghali AA, Xulu B, Revaprasadu N (2019). Synthesis and characterization of Z-scheme α-Fe_2_O_3_ NTs/ruptured tubular g-C3N4 for enhanced photoelectrochemical water oxidation. Sol. Energy.

[38] Mu H, Wan J, Wu Y, Xu J, Wang L, Cao X (2018). Novel polymer supported graphene and molybdenum sulfide as highly efficient cocatalyst for photocatalytic hydrogen evolution. Int. J. Hydrog. Energy.

[39] Palanivel B, Ayappan C, Jayaraman V, Chidambaram S, Maheswaran R, Mani A (2019). Inverse spinel NiFe_2_O_4_ deposited g-C_3_N_4_ nanosheet for enhanced visible light photocatalytic activity. Mater. Sci. Semicond. Process..

[40] Wang W, Shu Z, Zhou J, Li T, Duan P, Zhao Z (2018). Halloysite-derived mesoporous g-C_3_N_4_ nanotubes for improved visible-light photocatalytic hydrogen evolution. Appl. Clay Sci..

[41] Raza A, Shen H, Haidry AA, Cui S (2019). Hydrothermal synthesis of Fe_3_O_4_/TiO_2_/g-C_3_N_4_: advanced photocatalytic application. Appl. Surf. Sci..

[42] Du C, Huang H, Jian J, Wu Y, Shang M, Song W (2017). Enhanced electrocatalytic hydrogen evolution performance of MoS_2_ ultrathin nanosheets via Sn doping. Appl. Catal. A Gen..

[43] Lan Z-A, Zhang G, Wang X (2016). A facile synthesis of Br-modified g-C_3_N_4_ semiconductors for photoredox water splitting. Appl. Catal. B Environ..

[44] Li Y, Zhu S, Liang Y, Li Z, Wu S, Chang C (2020). Synthesis of α-Fe_2_O_3_/g-C_3_N_4_ photocatalyst for high-efficiency water splitting under full light. Mater. Des..

[45] Bai J, Xu H, Chen G, Lv W, Ni Z, Wang Z (2019). Facile fabrication of α-Fe_2_O_3_/porous g-C_3_N_4_ heterojunction hybrids with enhanced visible-light photocatalytic activity. Mater. Chem. Phys..

[46] Geng Y, Chen D, Li N, Xu Q, Li H, He J (2021). Z-Scheme 2D/2D α-Fe_2_O_3_/g-C_3_N_4_ heterojunction for photocatalytic oxidation of nitric oxide. Appl. Catal. B Environ..

[47] Pan J, Liu Y, Ou W, Li S, Li H, Wang J (2020). The photocatalytic hydrogen evolution enhancement of the MoS_2_ lamellas modified g-C_3_N_4_/SrTiO_3_ core-shell heterojunction. Renew. Energy.

[48] Han X, Xu D, An L, Hou C, Li Y, Zhang Q (2018). WO_3_/g-C_3_N_4_ two-dimensional composites for visible-light driven photocatalytic hydrogen production. Int. J. Hydrog. Energy.

[49] Li Y-P, Li F-T, Wang X-J, Zhao J, Wei J-N, Hao Y-J (2017). Z-scheme electronic transfer of quantum-sized α-Fe_2_O_3_ modified g-C_3_N_4_ hybrids for enhanced photocatalytic hydrogen production. Int. J. Hydrog. Energy.

[50] Yuan Y, Guo R-T, Hong L-F, Ji X-Y, Li Z-S, Lin Z-D (2021). Recent advances and perspectives of MoS2-based materials for photocatalytic dyes degradation: a review. Colloids Surf. A Physicochem. Eng. Asp..

